# Large volume 60 minute hyperthermia sonication with hilips Sonalleve V2 MR-HIFU System – animal in-vivo experiment

**DOI:** 10.1186/2050-5736-2-S1-A3

**Published:** 2014-12-10

**Authors:** Matti Tillander, Steffen Hokland

**Affiliations:** 1 MR-Therapy, Philips Healthcare, Finland

## Background

Mild hyperthermia (41.5-44°C) has been shown to enhance the effect of radiotherapy and chemotherapy. However, currently available heating methods are often inefficient in inducing conformal hyperthermia for deep seated targets. Here, the software of Philips Sonalleve V2 MR-HIFU system was modified to enable performing a long duration large volume hyperthermia sonication in in-vivo porcine thigh muscle.

## Materials and methods

The Sonalleve system was set to heat a circular area with a diameter of 45 mm at the depth of 5cm from the skin by combining electrical focal point steering with mechanical movement of the ultrasound transducer. The target temperature was set to 42.5°C and the heating duration was 60 minutes. The sonication was controlled using temperature maps in six slices (5 perpendicular and 1 along the beam axis, temporal resolution 3.2s and spatial resolution 2.5x2.5x7mm3) using proton resonance frequency MR thermometry. The unheated areas within the temperature maps were used for first order correction of the magnetic field drift.

## Results

The mean temperature in the 45mm target area reached 42.5°C approximately 10 minutes after starting of the heating. For the following 50 minutes the temperature was maintained resulting in an average mean temperature of 42.3°C for the time period. For the same area and time frame the corresponding average 10 and 90 percentile temperatures (T10 and T90) were 43.3°C and 41.3°C, respectively, indicating a very good temperature control.

The overall heated area had a shape of a cone reaching from the target area all the way to the skin. However, the energy deposition within the cone was lower in the near field than in the target area resulting in lower temperature elevation in the near field. The temperature mapping slice set in the near field showed average mean, T10, and T90 temperatures of 39.8°C, 41.0°C, and 38.2°C, respectively. The total heated volume exceeding 40°C was approximately 150cc, when estimating from the temperature slice aligned with the beam axis.

Immediately after the heating a contrast agent image using gadolinium was taken, which showed enhanced signal in the heated area indicating increased vessel permeability due to long duration hyperthermia.

## Conclusion

Clinical Philips Sonalleve V2 MR-HIFU system was used for conformal mild hyperthermia large volume heating at the depth of 5 cm for 60 minutes while keeping the temperature of the near field lower than at the target depth.

